# A Pathogenic Relationship of Bronchopulmonary Dysplasia and Retinopathy of Prematurity? A Review of Angiogenic Mediators in Both Diseases

**DOI:** 10.3389/fped.2018.00125

**Published:** 2018-06-13

**Authors:** Ashley Stark, Christiane Dammann, Heber C. Nielsen, MaryAnn V. Volpe

**Affiliations:** ^1^Tufts University School of Medicine, Boston, MA, United States; ^2^Division of Newborn Medicine, Department of Pediatrics, Floating Hospital for Children at Tufts Medical Center, Boston, MA, United States; ^3^Program in Cell, Molecular and Developmental Biology, Sackler School of Graduate Biomedical Sciences, Tufts University School of Medicine, Boston, MA, United States

**Keywords:** bronchopulmonary dysplasia, retinopathy of prematurity, vasculogenesis, prematurity, angiogenesis

## Abstract

Bronchopulmonary dysplasia (BPD) and retinopathy of prematurity (ROP) are common and significant morbidities of prematurely born infants. These diseases have in common altered and pathologic vascular formation in the face of incomplete organ development. Therefore, it is reasonable to question whether factors affecting angiogenesis could have a joint pathogenic role for both diseases. Inhibition or induced expression of a single angiogenic factor is unlikely to be 100% causative or protective of either of BPD or ROP. It is more likely that interactions of multiple factors leading to disordered angiogenesis are present, increasing the likelihood of common pathways in both diseases. This review explores this possibility by assessing the evidence showing involvement of specific angiogenic factors in the vascular development and maldevelopment in each disease. Theoretical interactions of specific factors mutually contributing to BPD and ROP are proposed and, where possible, a timeline of the proposed relationships between BPD and ROP is developed. It is hoped that future research will be inspired by the theories put forth in this review to enhance the understanding of the pathogenesis in both diseases.

## Introduction

Premature infants are at risk of developing bronchopulmonary dysplasia (BPD) and retinopathy of prematurity (ROP) that despite advances in neonatal care and neonatal research, remain significant causes of morbidity and mortality in preterm infants. BPD is characterized by altered alveolarization and reduced and abnormal alveolar microvascular development and remodeling in the premature lung (1). ROP is also a disease of altered vascular development, occurring in the premature retina (2). Both BPD and ROP are associated with significant alterations in cell signaling that impact the regulation of angiogenesis (1, 2). Frequently infants develop both diseases. BPD and ROP also share numerous physiologic risk factors in their pathological development (1, 2). These facts suggest that molecular pathways common to both BPD and ROP are likely. While a balance between pro-angiogenic and anti-angiogenic signaling is important for controlled neoangiogenesis and vascular remodeling (3), little is known about this balance in either BPD or ROP development. This review focuses on the commonalities between BPD and ROP in the dysregulation of pro- and anti-angiogenic factors involved in vascular development of the lung and retina. The goal is to assemble evidence that the two diseases may have common or closely related mechanistic pathways of pathologic development, even though clinical signs may present at different times. There are no current animal models that address the pathogenesis of both diseases together, nor has this topic been discussed in detail in the literature. Targeting angiogenic factors common to both diseases may be important to future research studies and a useful therapeutic strategy to prevent pathologic angiogenesis in both the lung and the retina.

**Figure 1:**
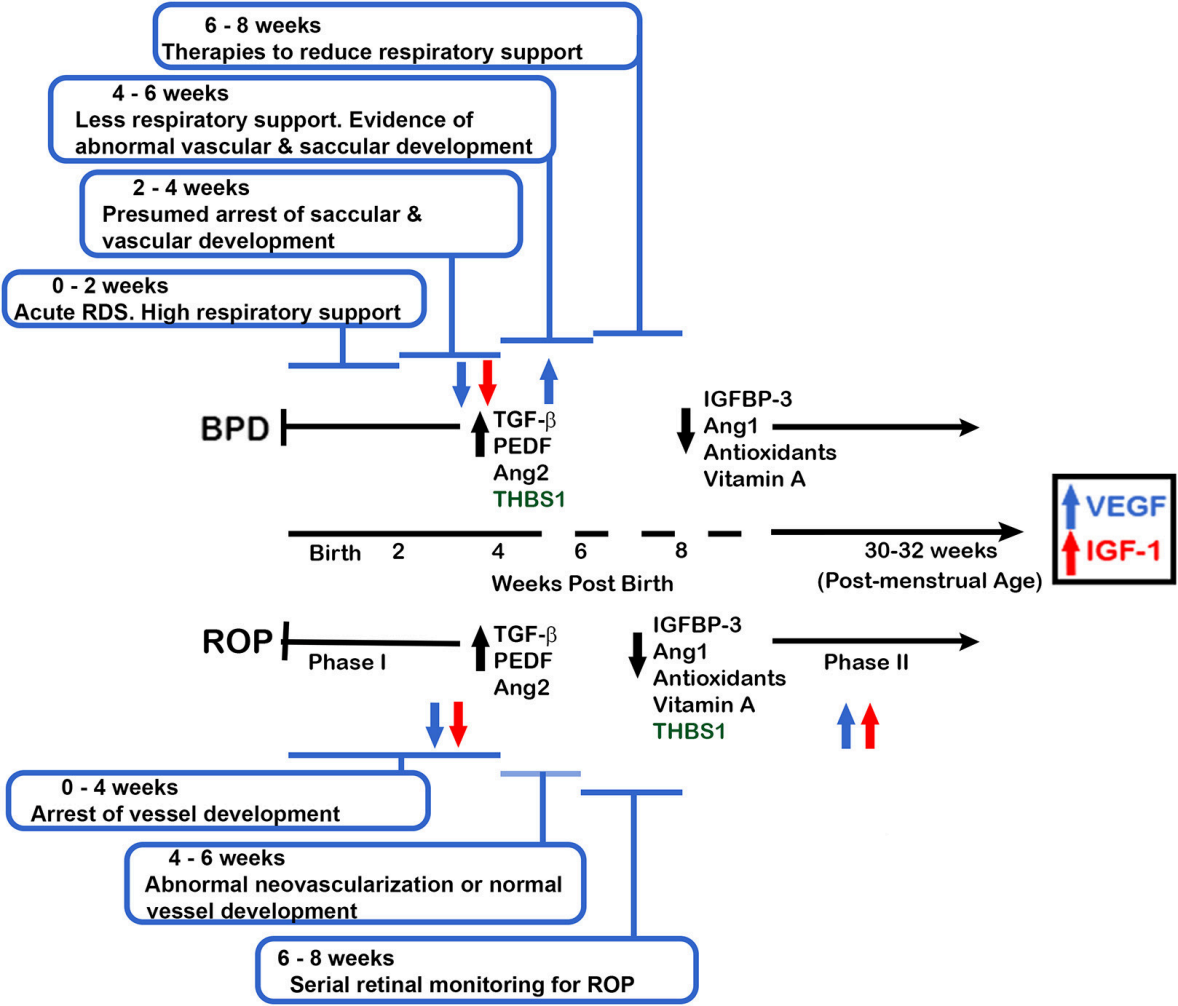
Proposed clinical timeline depicting the current understanding of BPD and ROP disease development. The data are coordinated with the changes in expression and function of the vascular factors common to BPD and ROP. Using a gestational age of 24 weeks at birth, and current BPD and ROP clinical symptoms and definitions, an infant is determined to have or not have BPD at 36 weeks PMA (approximately 12 weeks of age) but will have clinical signs of developing BPD prior to that time. Phase 1 ROP occurs prior to 31 weeks PMA whereas Phase 2 (development of neovascularization) is believed to occur at 30-32 weeks PMA (6–8 weeks of age for an infant born at 24 weeks gestation). Abbreviations are as documented in text.

It is likely that cellular changes that cannot be documented using current diagnostic tools take place in the premature lung and retina before the point in time when BPD and ROP can be clinically diagnosed. These early events may provide clues to the existence of molecular pathways common to the pathological course of both BPD and ROP and identify common mechanisms in their initiation and/or progression. This review focuses on mediators of angiogenesis important for lung and eye development and the pathogenesis of BPD and ROP. Most of the work in this field addresses promoters of angiogenesis. However, inhibitors of angiogenesis are also important in development and disease and these are also reviewed. Table 1 lists the vascular mediators that will be discussed in this review. A side-by-side timeline of the current understanding of clinical events in the development of BPD and ROP is shown in Figure 1. This figure also incorporates the timing at which mediators of vascular development discussed in this review are proposed to be active in BPD and ROP. Both the timeline and the table are referred to throughout the review.

**Table 1:** List of vascular factors common to BPD and ROP discussed in this review.

**MEDIATORS OF NEOVASCULARIZATION**		
**Factor**	**Role in neovascularization**	**References**
VEGF	Stimulates development of blood vessels	(1, 2, 15–27)
sFlT-1	Endogenous VEGF antagonist	(25, 26)
VEGF Polymorphisms	Increase circulating VEGF activity	(16, 19, 29–31)
eNOS and NO	eNOS regulates NOS production, which consequently regulates endothelial progenitor cell differentiation, apoptosis, and differentiation at vessel formation sites	(1, 15, 33, 37–44)
IGF-1	Promotes growth throughout the body	(2, 45–50)
Interferon	PAPP-a inhibitor	(48, 50)
IGFBP-3	Major regulator of free IGF-1 levels	(28, 51–53)
Angiopoietins	Regulators of neovascularization, influenced by VEGF	(13, 18, 54–57)
Tie2 and soluble Tie2 receptors	Promotes endothelial cell survival and maturazion and vessel stabilization	(13, 18, 55)
TGF-β	Regulator of cell growth, differentiation, migration, and extracellular matrix protein production	(13, 22, 46, 58–71)
Superoxide dismutase	Prevent cell injury by catalyzing reduction of superoxide radicals	(72–79)
Vitamin A	Influences endothelial cell proliferation required for angiogenesis	(80–86)
Trombospondin-1	Ca binding extracellular glycoproteins with anti-angiogenic properties that regulate cell-cell and cell-matrix interactions	(13–87)
PEDF	Neuroprotective growth factor with anti-angiogenic properties	(88–98)

*Abbreviations used in Table are as noted in text*.

BPD and ROP share the major risk factors of perinatal inflammation and exposure to oxidative stress (1, 2, 4–6). Additionally, although from a genetic standpoint BPD and ROP are not considered independent predictors of each other, both diseases have a strong genetic predisposition (7, 8). However, the specific genetic contributions have not yet been fully elucidated. Further, while the observed developmental sequence and timing of BPD and ROP are clinically well-documented (Figure 1), it is not clear if the temporal development at the cellular level is similar. This is partially due to the inability to view the lung's pathological changes in real-time, whereas vascular changes in the developing eye are easily tracked by visual inspection. Due to the inability to view the lung's pathology non-invasively, BPD is currently diagnosed by gestational age and treatment criteria rather than specific clinical biomarkers of lung pathology (9). Physicians must be aware that these criteria do not capture all patients with respiratory problems associated with lung immaturity and injury. Recently, experts have called for a revision or refinement of the current definition of BPD to better reflect the spectrum of at-risk infants, including those with abnormalities of respiratory control and of the airways (10–12).

Abnormal retinal vessel development after preterm birth occurs in a biphasic pattern in ROP (Figure 1). The first phase of ROP involves altered retinal neovascularization with cessation of new vessel growth and regression of existing vessels, while the second phase is characterized by pathological vessel growth and neovascularization in response to retinal hypoxia (2). This biphasic process is easily seen in living infants with ROP by retinal examination. On the other hand, documentation of normal and pathologic vascular changes in the lungs of live infants requires invasive procedures that are not justifiable. However, the work of De Paepe et al. using autopsy specimens provides evidence for a biphasic pattern of blood vessel development and maldevelopment in BPD like that of ROP, contrary to a simple monophasic developmental arrest proposed by others (4, 13, 14). The time line shown in Figure 1 utilizes this biphasic model for vascular changes in ROP and BPD, and is displayed using gestational and post-natal age to correlate clinical and investigative data.

## Vascular endothelial growth factor

Numerous studies show that vascular endothelial growth factor (VEGF) dysregulation has a major role in both BPD and ROP pathogenesis (1, 2, 15–21). VEGF is decreased in the early post-natal period of infants born during the late canalicular and early saccular stages of lung development, when progression of lung vascular and airway development is essential for adequate alveolar and microvascular formation and function. Been et al. found significantly decreased VEGF levels in tracheal aspirates at days of life 0 and 3 from infants born at 28–29 weeks gestation who later developed BPD, correlating early decreases in lung VEGF protein levels prior to BPD recognition (22). Interestingly, these same infants (with altered VEGF levels and BPD) were also receiving more supplemental oxygen support compared with those who did not develop BPD. Supplemental oxygen given to premature infants to support respiratory difficulties is also associated with a premature decrease in normal retinal VEGF production (16). This pathologic decrease in retinal VEGF occurs in phase I of ROP (from the time of premature birth to approximately 30–32 weeks of gestation) and is more likely to occur with decreasing gestational age at birth (2, 17). This time for phase 1 of ROP may correspond to the timing in the lung where recent research shows a down regulation of VEGF (Figure 1). However, in phase II of ROP, beginning at 30–32 weeks post-menstrual age, local retinal hypoxia with further retinal growth leads to resurgence of retinal VEGF that drives abnormal retinal vessel formation (17).

This change from decreased to elevated VEGF expression in ROP has also been demonstrated by Bhandari et al. and others in some animal models of oxygen-induced lung injury and in human studies (23, 24). In tracheal aspirates from infants who went on to develop BPD or died, VEGF levels were initially increased (first 12 h of life) followed by a decrease and then became increased again by day 21–28 (23). Although this study and others used oxygen levels much higher than usually needed in current clinical practice, it raises the question of whether the biphasic change of lung VEGF levels seen in some animal models and in human studies corresponds to the biphasic VEGF response that occurs in infants with ROP (17, 23, 24). However, studies in humans where BAL levels of VEGF have been measured may not correspond temporally in lung development to when BAL VEGF levels were measured in the animal study of Bhandari and colleagues (21–24).

Endogenous VEGF antagonists are also coming to attention as potential contributors to the development of BPD. The endogenous VEGF antagonist soluble fms-like tyrosine kinase-1 (sFlt-1) is present in amniotic fluid and is markedly increased with chorioamnionitis and pre-eclampsia, where it mediates maternal pregnancy-related vascular disease. Postulating that sFlt-1 from pregnancy participates in the development of BPD in the premature infant, Wallace et al. (25) developed rat models to study this. Injection of sFlt-1 into the amniotic sac of rat fetuses created BPD-like changes in neonatal lung alveoli, lung function, and evidence of right heart hypertrophy. Injection of endotoxin into the rat amniotic fluid created a model of chorioamnionitis in which sFlt-1 levels were elevated in the neonatal rat pups. These pups developed evidence of BPD in the first days of life. The changes of BPD were prevented by administration of an antibody against sFlt-1 into the amniotic fluid. This recent work indicates the possibility of an antibody treatment to prevent BPD in premature infants exposed to chorioamnionitis before birth (26).

Given the essential need for regulated VEGF expression in both ROP and BPD, investigators have expressed the concern that treatment of ROP with intra-vitreous injection of anti-VEGF therapy may adversely impact lung vascular development via unintended systemic side effects (27). Further, there is increasing concern that anti-VEGF therapy is being used to treat ROP in ways that were not studied in clinical trials. This could further complicate an interplay between VEGF expression and VEGF related therapies in ROP and BPD (28). More research is warranted to further elucidate the exact timeline with which VEGF affects angiogenic development in both BPD and ROP and what consequences may arise at certain time points with anti-VEGF therapy.

## VEGF polymorphisms

Some studies have addressed VEGF single nucleotide polymorphisms (SNPs) as a risk factor for BPD or ROP. One candidate SNP is the VEGF 460C/T allele on chromosome 6. Haplotypes containing this C
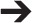
T polymorphism had a 71% increase in promoter activity, suggesting that a consequential increase in VEGF activity would be observed (19). While no increase was detected in baseline VEGF or in response to stimuli, carriage of the −460T allele was an independent risk factor for BPD development, at approximately 9% above baseline risk. In contrast, neonates homozygous for the −460C allele were at the lowest risk of developing BPD. An increased proportion of infants with this same −460C
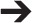
T allele developed ROP requiring treatment, yet increased serum VEGF levels were not seen. This suggests that local VEGF expression may be a more important biomarker than circulating VEGF levels (29). In another study, after adjustment for gestational age, supplemental oxygen therapy and gender, the VEGF −460TT/+405CC haplotype was associated with an increased likelihood and the −460TT/+405GG haplotype with a decreased likelihood of requiring ROP treatment (16). The authors suggested that the correlation of genetic variants in VEGF genotypes with the outcomes of ROP and ROP requiring treatment may be due to linkage disequilibrium with nearby angiogenic genes with unknown genetic variants. Additionally, carriage of the −634C>G allele has been shown to independently increase the risk of BPD and ROP in Japanese and Egyptian neonates, respectively, while the 936CT allele did not (30, 31). Although these studies focused on BPD and ROP individually, comparison of these studies highlights the importance of reviews to synthesize research across multiple disease processes. Together, these studies on VEGF levels and VEGF polymorphisms suggest that local tissue VEGF levels in the retina and broncho-alveolar regions are likely more meaningful for disease causation than circulating VEGF levels, and that plasma or tracheal aspirate VEGF levels are likely much less sensitive predictors of disease risk. Similar conclusions of local tissue vs. circulating biomarkers were recently documented for asthma (32). These VEGF studies also help validate the importance of a multi-site study of the role of VEGF polymorphisms in the concomitant development of BPD and ROP.

## Endothelial nitric oxide synthase

**Figure 2:**
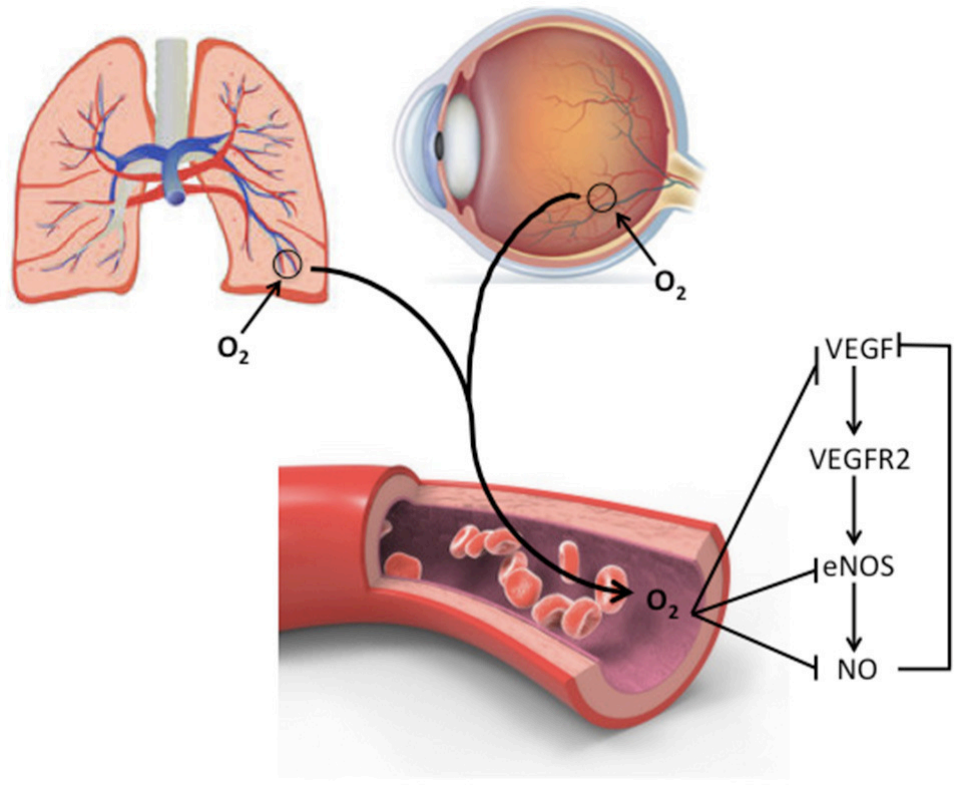
Interaction of O_2_ with VEGF and eNOS signaling. Oxygen can disrupt VEGF and NO signaling pathways via down regulation of eNOS. Abbreviations are as documented in text.

One of the effects of altered VEGF levels in BPD and ROP is the impact on activation of the type 2 VEGF receptor (vascular endothelial growth factor receptor 2, VEGFR2) that stimulates endothelial nitric oxide synthase (eNOS) production in endothelial cells (33). ENOS is responsible for nitric oxide (NO) production, which subsequently regulates endothelial progenitor cell proliferation, apoptosis, and differentiation at vessel formation sites (33). NO also serves as a vasodilator by relaxing smooth muscle cells, facilitating blood flow, and vascular tonicity. It also prevents platelet aggregation (34). Oxygen exposure (40–50%) significantly reduced eNOS and NO levels in endothelial colony forming cells isolated from preterm infant cord blood. The authors concluded that in the development of BPD, oxygen-induced decreases in VEGF disrupt the VEGF-NO signaling pathway, which is important for vascular and alveolar growth (Figure 2) (33). Consistent with these findings, VEGFR inhibition in neonatal rats decreased eNOS expression and thus NO production (1). Inhaled NO can overcome the effects of simultaneous prolonged VEGFR inhibition by preventing lung endothelial apoptosis, improving vascular growth and enhancing alveolarization (1, 33). In contrast, in a mouse model of ROP, both vaso-obliteration and vaso-proliferation were significantly decreased by eNOS blockers and in eNOS null mice (35). Both eNOS −/− and eNOS +/+ mice exposed to 75% oxygen between P7 and P9 showed a decline in VEGF production. However, this decline of VEGF in eNOS −/− mice was 30% as opposed to 77% in eNOS +/+ mice. ENOS −/− mice also had significantly less extraretinal neovascularization (36). At least in the eye, NO and VEGF exhibit feedback regulation, as VEGFR2 activation by VEGF promotes eNOS activity leading to NO formation and subsequent down regulation of VEGF (36) (Figure 2). This difference in eNOS regulation in lung and retinal development highlights the importance of considering potential effects on both organs when considering treatments that alter VEGF signaling. Although a treatment that would decrease eNOS activity in the eye may attenuate ROP development, systemic side effects may negatively impact lung blood vessel development.

Despite the optimism in rat and human *in vitro* studies evaluating NO treatment, meta-analyses of studies using inhaled NO therapy in preterm neonates for prevention or treatment of BPD show a lack of benefit (15, 37–41). A major limitation of most of these meta-analyses was the lack of evaluation of potentially influential factors including gender, gestational age, ethnic group/race, socioeconomic status, and modes of ventilation. Additionally, the 14 trials considered in these meta-analyses were not comparable due to differences among centers in other standard therapies and treatment strategies. The timing of the NO treatment was also quite variable, ranging from early routine, early rescue, and later rescue, which further limited the value of the meta-analyses (37). Askie et al. attempted to control for patient and treatment characteristics by doing an individual patient analysis using data from many of the major NO trials (15). Instead of combining aggregate data from each trial, they attempted to ensure uniformity in defining patient characteristics, outcomes, and treatments by using the central repository of “raw data” from each randomly assigned subject. However, despite this rigorous analysis of data from 11 trials, NO still did not exhibit a beneficial effect on BPD and is not currently recommended to prevent or treat this disease. This meta-analysis did not address the incidence of pulmonary hypertension in BPD, which is increasingly recognized as a significant complication of BPD (42, 43). Thus, the value of NO administration to prevent or treat the development of pulmonary hypertension complicating BPD remains controversial. Of interest to our current review, Askie et al. did include severe ROP as a secondary outcome, finding no protective effect of NO against severe ROP. More research is needed to understand the relationship between NO regulation and the development of BPD and ROP. Future studies should also control for potential contributing factors such as race, mode of respiratory support, illness severity and timing of therapeutic interventions (42, 44). The most recently completed multicenter trial showed that while use of NO therapy from 5 to 14 days of age was safe, it did not improve survival with BPD at 36 weeks postmenstrual age. The authors suggested that an unbalanced racial distribution between the placebo and NO groups may have been a clinically relevant factor (44).

## Insulin like growth factor-1 and interferon γ

The fetus receives insulin like growth factor 1 (IGF-1) via the placenta and amniotic fluid. IGF-1 serum concentration increases significantly throughout the third trimester. Preterm birth then prevents this normal third trimester rise in serum IGF-1, and IGF-1 continues to drop with extra-uterine advancing gestational age (45, 46) (Figure 1). Lower serum levels of IGF-1 are associated with both BPD and ROP development (47). In contrast, tissue samples taken at autopsy from preterm infants with established BPD showed that lung tissue IGF-1 levels were greatly increased compared to lungs of infants who died shortly after birth. The increase was especially prominent in the epithelial and mesenchymal compartment (46). The difference in serum vs. diseased lung tissue IGF-1 levels in established BPD is possibly due to local inflammation-induced production of IGF-1 by activated alveolar macrophages and monocytes (46, 48).

A slower rise in serum IGF-1 levels in the first 4 weeks following premature birth is an independent risk factor for ROP (2). In phase I ROP, low postnatal IGF-1 levels may contribute to the abnormal vascular development while in phase II, increasing IGF-1 levels with advancing post-conceptual age may promote abnormally maximized stimulation of VEGF, contributing to pathogenic neovascularization (2). It is possible that early supplementation of IGF-1 in the newborn infant could attenuate the development of phase I, which may prevent the induction of phase II (2).

**Figure 3:**
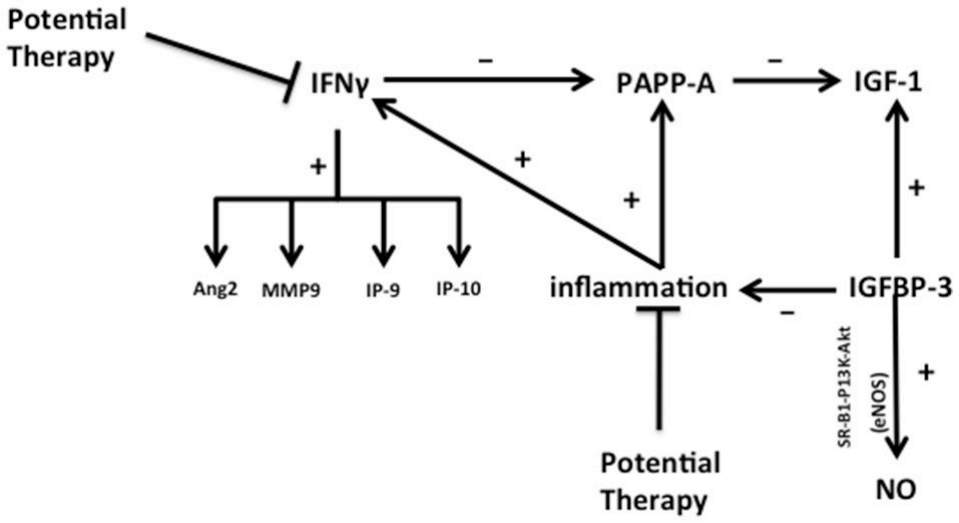
The regulation of inflammation during lung and retina injury in BPD and ROP. The complex interplay of major factors (PAPP-A, IFNγ, IGF-1, IGFBP-3, and NO) in promoting or reducing inflammation and tissue damage is illustrated, and potential targets for therapeutic intervention are identified. IGFBP-3 upregulates NO in both lung microvascular and hematopoietic stem cells (through PI3K-Akt signaling) and promotes IGF-1 activity, thereby decreasing inflammation and endothelial cell death. In addition to nutritional-induced increased IGFBP-3 levels, the interplay of pathways shown here suggests other points for possible direct therapy. For example, direct inhibition of inflammation and IFNγ may inhibit PAPP-A and release IGF-1 production. Targeting IFN may decrease the expression of Ang2, MMP9, IP-9, and IP-10. Abbreviations are as documented in text.

Serum free IGF-1 levels are impacted by pregnancy associated plasma protein A (PAPP-A), a metalloproteinase that enhances dissociation of IGF-1 from insulin-like growth factor binding proteins 2, 4, and 5 (IGFBP-2, 4, and 5) increasing bioavailable IGF-1 (48) (Figure 3). PAPP-A is produced by fibroblasts, endothelial and smooth muscle cells, with hypoxic, oxidative, and inflammatory stresses increasing its bioactivity (49). This demonstrates how inflammation can play a vital role in systemic control of free IGF-1 levels. Decreased tissue IGF-1 could be accomplished through decreasing the level of inflammation and oxidative stresses in the lung or by decreasing the levels of interferon gamma (IFNγ), a PAPP-A inhibitor (48). Inflammation can also increase IFNγ levels, which increases angiopoietin 2 (Ang2), matrix metalloproteinase 9 (MMP9), interferon γ-inducible 10-kDa proteins 9 (IP-9) and 10 (IP-10), all of which are downstream targets of IFN-γ, and could be potentially interpreted as markers for the development of BPD and ROP (50).

## Insulin like growth factor binding protein 3

Insulin-like growth factor binding protein 3 (IGFBP-3) is a major regulator of free IGF-1 levels. It also has independent IGF-1-like activity. Figure 3 depicts pathways influenced by IGFBP-3 that may coordinate different protective responses to lung or retinal injury that induce abnormal vascular remodeling. The figure also shows specific points that have commonly been considered as therapeutic targets. Like IGF-1, serum levels of IGFBP-3 increase with advancing *in utero* gestational age but can decrease postnatally after preterm birth (51). IGFBP-3 promotes the eNOS-mediated increase of NO in lung microvascular endothelial cells and hematopoietic progenitor stem cells via the SR-B1—PI3K—Akt signaling pathway (52) linking the pathways depicted in Figure 3 with those discussed in relation to Figure 2. IGFBP-3 reduces endothelial cell death and inflammation, reducing the production of IFNγ and its downstream inflammatory mediator targets. IGFBP-3 also increases astrocyte—endothelial cell interactions, resulting in enhanced neurovascular coupling and barrier properties (52). This effect of IGFBP-3 likely leads to a decrease in vessel permeability and an increase in maturation of blood vessels (52). IGFBP-3 levels are influenced by nutritional status and correlate positively with weight change and protein intake in infants with and without BPD (51). Adequate nutrition in premature infants promotes normal lung development and growth, immune system function, oxygen tolerance, and enhances lung repair following injury (51).

Premature infants with established ROP have lower serum IGFBP-3 levels than infants with no ROP (52). IGFBP-3 deficient mice exposed to oxygen for 5 days had increased retinal vessel loss compared to controls (53). Retinas of mice injected with IGFBP-3 and exposed to 75% oxygen showed significantly reduced endothelial cell death in the mid-peripheral and peripheral retina in both phases of ROP (52).

Overall these studies suggest that a systemic increase in IGFBP-3, potentially achieved through improved nutrition, could prevent or slow the progression of BPD by increasing eNOS - mediated NO production in lung microvascular endothelial cells, by downregulating inflammation and reducing IFNγ and activation of IFNγ targets, while promoting IGF-1 beneficial effects (Figures 2, 3). Similarly, IGFBP-3 may decrease the progression of ROP by reducing retinal vaso-obliteration and abnormal vaso-proliferation. Unfortunately, ongoing Phase 1 and Phase 2 trials evaluating the use of recombinant IGF-1/IGFBP3 in the prevention of ROP have so far failed to show a difference in severity of ROP but may have shown a trend toward less severe BPD in the treatment group. However these trials were not powered to evaluate BPD incidence or severity (28) (NCT 01096784). In addition to IGF-1/IGFBP3 interactions, defining how the regulatory mediators IFNγ, PAPP-A, Ang2, MMP9, IP-9, and IP-10 intersect in BPD and ROP may promote the development of new therapies that simultaneously target the development of both diseases while enhancing or allowing normal development to progress (Figure 3).

## Angiopoietins

**Figure 4:**
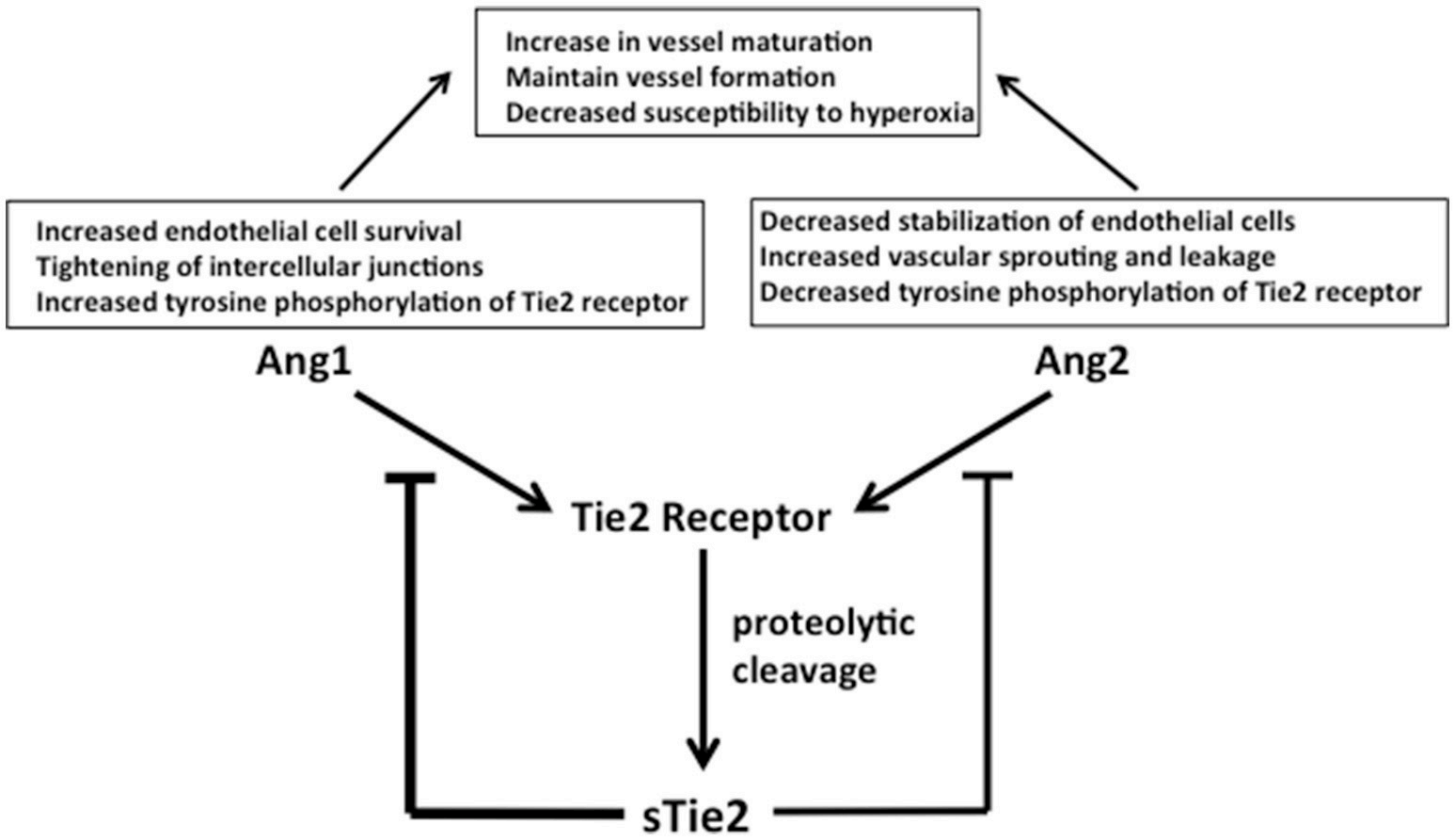
Ang1 and Ang2 function and regulation. Ang1 and Ang2 control competitive regulatory events in the vascular endothelium. Competitive binding to their common receptor, Tie2, regulates endothelial cell stability and function to inhibit or promote neoangiogenesis, respectively. Ang1 increases endothelial cell survival, tightens intercellular junctions, and increases tyrosine phosphorylation of membrane-bound Tie2. Ang2 decreases endothelial cell stability, increases vascular sprouting and leakage, and decreases tyrosine phosphorylation of Tie2, promoting angiogenesis. The resulting competitive signals combine to stabilize formed vessels, allowing them to mature, and simultaneously maintain neovascularization as dictated by tissue oxygen needs. The amounts of free Ang1 and Ang2 available for binding Tie2 are modulated by soluble Tie2 (sTie2), which is formed by proteolytic cleavage and release of the extracellular component of the membrane bound Tie 2 receptor. Circulating sTie2 binds both Ang1 and Ang2, making them unavailable for binding Tie2 to influence endothelial cell functions. Because sTie2 has a higher affinity for Ang1 than Ang 2 the amount of sTie2 exerts differential control of Ang1 and Ang2 effects. Increased levels of sTie2 favor an overall increase in new vessel sprouting because more Ang1 than Ang2 is bound up. Abbreviations are as documented in text.

Angiopoietins are critical modulators of physiologic and pathologic neovascularization. Their production is intimately connected with that of VEGF (54). Both angiopoietin (Ang)1 and Ang2 interact with the Tie2 receptor whose expression is largely restricted to endothelial cell membranes (18). Ang1 binding to Tie2 induces tyrosine phosphorylation, promoting endothelial cell survival and maturation and tightening of endothelial cell intercellular junctions for vessel stabilization (Figure 4). Tie2 levels were significantly downregulated in fetal lung autopsy samples from ventilated infants between 25 and 27 weeks post-menstrual age, suggesting decreased Ang1 signaling (13). Ang1 interaction with VEGF is critical for normal vascular maturation. Therefore, it is interesting that VEGF and Ang1 administration as combined gene therapy to neonatal rats stimulated lung growth and vascular maturation more effectively than VEGF therapy alone (18).

Ang2 counteracts Ang1 by decreasing tyrosine phosphorylation of Tie2. This promotes destabilization of endothelial cell junctions to enable both vascular leakage and sprouting of new vessels (18, 55) (Figure 4). Ang2, therefore, can also interact with VEGF to stimulate angiogenesis under hypoxic conditions, however Ang2 can cause endothelial cell death and vessel regression in the absence of sufficient pro-angiogenic signals (55) linking the mechanisms addressed in Figure 4 with those shown in Figure 2. This latter effect of Ang2 in the presence of insufficient pro-angiogenic signaling is a potential scenario in BPD and ROP.

The temporal patterns of Ang1 and Ang2 expression levels in BPD and ROP are similar (Figure 1). Ang1 is decreased in infants with established BPD and in ROP of increasing severity, while Ang2 is increased in the tracheal aspirates of infants with established BPD and in the vitreous fluid of eyes with progressing ROP (54, 55). Infants born at less than 30 weeks gestational age who subsequently developed moderate BPD or died with symptoms suggesting developing BPD had significantly higher Ang2 in tracheal aspirates as early as 1 and 7 days of life and as late as day 10 of life in some studies (55, 56). Among infants with ROP who were an average gestational age at birth of 24.2 weeks, those with stage 4 ROP and mild to high neovascularization activity had significantly higher vitreous levels of both Ang1 and Ang2 compared to control infants. However, the degree of elevation of Ang2 was much greater than Ang1 (54). These studies demonstrate that in both BPD and ROP the ratio of Ang1 to Ang2 favors the anti-angiogenic Ang2; this imbalance may contribute to abnormal vessel formation in both the lung and the eye. While this abnormal vessel development cannot be documented within the living human lung, the work by DePaepe et al. suggests that like the immature retina, the lung also undergoes abnormal, and attenuated vessel formation after preterm birth (57).

Endothelial cells have the capacity to alter the balance of activity between Ang1 and Ang2. Proteolytic cleavage of the extracellular domain of Tie2 releases soluble Tie2 (sTie2) from the endothelial cell membrane (Figure 4). Binding of sTie2 to free Ang1 and Ang2 prevents these growth factors from binding intact membrane bound Tie2, altering endothelial cell function in vascularization. sTie2 has a higher affinity for Ang1 (55) (Figure 4). Therefore, high sTie2 concentrations could shift the Ang1/Ang2 ratio to favor Ang2 action, contributing to systemic and pulmonary endothelial cell activation and neoangiogenesis (55).

It is possible that decreasing sTie2 concentrations by inhibiting the proteolytic cleavage of the extracellular domain would be beneficial in the treatment of BPD and ROP. This would shift the Ang1/Ang2 ratio toward Ang1 activity, driving stabilization of developing vessels while maintaining neovascular formation via Ang2, thereby reducing susceptibility to oxygen effects on vessel development. Finally, higher intra-amniotic levels of Ang2 were found in women presenting with intra-amniotic inflammation, suggesting that decreasing inflammation during pregnancy could also reduce Ang2 activity in the premature infant (55) (Figures 3, 4).

## Transforming growth factor β

The transforming growth factor beta (TGFβ) family members signal through dimers of TGFβ type I and II receptors, and are vital for regulation of cell growth, differentiation, migration, and extracellular matrix protein production, events that all play important roles in the developing fetus and newborn, including the regulation of vascular development (58). A large body of research has addressed the role of TGFβ in lung development and disease. In early lung development, TGFβ participates in the control of airway branching morphogenesis, although excessive TGFβ inhibits lung branching (46, 59). Later in lung development TGFβ inhibits alveolar type II cell maturation (60, 61). Chronic hyperoxia in rat lungs leads to a significant increase in active TGFβ levels in BAL and a significant upregulation of the TGFβ receptors I and II (59). Overexpression of TGFβ1 in neonatal rats decreases alveolarization whereas physiologically normal TGFβ levels decrease apoptosis, promote alveolar type II cell repair after exposure to hyperoxia and support expression of alpha smooth muscle actin (αSMA), suggesting increased capillary maturation with less permeability. Recent research by Sureshbabu et al. suggests that TGFβ-mediated apoptosis, inflammation, reduced alveolarization and impaired angiogenesis in a BPD mouse model is via the type II TGFβ receptor (TGFβR2) (62). Ventilated preterm infants also have elevated tracheal aspirate levels of TGFβ1 in both its latent and active forms compared to non-ventilated age-matched infants (22). However, TGFβ1 biology in the lung is complex and often appears contradictory. For example, TGFβ1 enhances survival of rat alveolar type II cells in culture (63).

Type 1 and type II receptors are present on vascular endothelial cells and pericytes of the developing rodent retinal vasculature, suggesting that TGFβ receptor regulation directly modulates retinal vascular development (58). Shih et al. evaluated the relationship of TGFβ1 expression and susceptibility to hyperoxia-induced retinal vascular changes in bovine retinal endothelial cells and in neonatal mice. Vascular endothelial growth factor receptor 1 (VEGFR1), a critical factor for vessel survival, was increased 6.5-fold compared to controls in bovine retinal endothelial cells exposed to TGFβ1. The authors went on to study neonatal mice exposed to 24 h of hyperoxia (FiO_2_ 0.75). This degree of hyperoxia reduced the levels of retinal TGFβ1 and VEGFR1 by one third. Administration of TGFβ1 following 17 h of hyperoxia exposure protected retinal vascular development. These TGFβ1-treated mice retained more retinal capillaries than controls. TGFβ1 also induced retinal pericyte αSMA expression. Pericytes in the retinal vascular bed express αSMA. Specifically, pericytes of mature retinal vessels all express αSMA, but only a fraction of vascular pericytes express αSMA while neovascularization of the retina is in process. It is important to note that retinal vessels whose pericytes express αSMA are resistant to oxygen-induced vessel loss (64, 65). The work of Shih et al. suggests that induction of VEGFR1 and αSMA by TGFβ1 may protect the premature retina from hyperoxia-induced inhibition of neovascularization, the first event in the development of ROP (Figure 1). αSMA is an important factor for stabilization of new vessels (64, 65). In ROP, vessel maturation may be delayed or attenuated by the lack of VEGFR1 due to decreased pericyte expression of TGFβ1 (64). In contrast, other work found that the angiogenic effect of TGFβ was accompanied by the presence of an inflammatory cell infiltrate instead of positive regulation of VEGFR1 (58). The authors of this study suggested that the presence of TGFβ in the retina is secondary to the inflammatory response induced by hyperoxia, as TGFβ did not stimulate angiogenesis in the absence of inflammation (66).

Endoglin is a hypoxia inducible accessory receptor for TGFβ expressed predominantly in proliferating vascular endothelium. It is significantly up regulated in the microvasculature of post mortem lung tissue from ventilated preterm infants (13, 67). Endoglin biology, like TGFβ, is also complex. It exists in the canonical membrane-bound form and as a shorter soluble form that binds TGFβ and exerts effects that can be the opposite of the membrane bound form. Fetal rat pups given a vector expressing soluble endoglin via intraamniotic injection at embryonic day 14 had significantly elevated levels of endoglin in the lungs at 2 weeks of age. The lungs had arrested development of the microvascular bed and the alveolar structures, similar to human BPD (68, 69).

Endoglin is also a mediator of ROP development. Neonatal mice exposed to hyperoxia during the first week of life develop neovascular retinopathy that models human ROP. However, neonatal mice that are haploinsufficient for endoglin have markedly reduced neovascular retinopathy under the same conditions (70). A neonatal rat hyperoxia model of ROP confirmed that reducing endoglin in the retinal vasculature (using antibody treatment) blocked the neovascularization response to hyperoxia. The same effect was seen when VEGF was blocked by antibody treatment (71). This study confirmed that VEGF, a major activator of neovascularization in ROP, requires elevated levels of endoglin to promote neovascularization.

Overall, the animal and human studies suggest complex involvement of TGFβ signaling in BPD and ROP pathophysiology. Additional research should likely involve more focus on endoglin, α-SMA, and VEGFR-1 to more clearly elucidate the role of TGFβ. Since physiologic levels of TGFβ1 appear to have a positive effect on pulmonary and retinal vessel development, more research that focuses on maintaining physiologic TGFβ levels in preterm neonates may be a valuable strategy for developing new therapeutic approaches.

## Superoxide dismutase

Recent work provides increasing evidence that levels of antioxidants are important for protecting normal angiogenesis in the lungs and eyes. Fetal antioxidant levels increase *in utero* during the third trimester. Thus, since the infants at greatest risk of developing BPD or ROP are born early in the third trimester they necessarily have low antioxidant levels and are more sensitive to cellular damage by reactive oxygen species (72, 73). Superoxide dismutases (SOD) are a family of proteins that prevent cell injury by catalyzing the reduction of the superoxide radical (${\text{O}}_{2}^{-}$) produced during hyperoxia or reperfusion. Mammals have three types of SOD proteins, SOD1 or cytoplasmic SOD, SOD2 or mitochondrial SOD, and SOD3 or extracellular SOD. Both SOD1 and SOD3 require the trace elements Zn and Cu as cofactors (CuZn-SOD); levels of SOD3 are more closely associated with BPD and ROP than the other types of SOD (74, 75). Transgenic mice in which the coding sequence for SOD3 was removed developed and aged normally. Despite loss of SOD3 protein they did not have increased levels of SOD1 and SOD2 proteins. These mice had significantly more lung damage and reduced survival following exposure to 1.0 FiO_2_ compared to mice with a normal extracellular SOD3 gene (76). Although supplementation of very premature human infants with recombinant human CuZn-superoxide dismutase (rhSOD3) did not reduce the incidence of BPD, long term follow-up studies showed a significant decrease in the incidence of chronic lung disease in supplemented infants (generally manifested as reactive airway disease) (73, 74, 77). Twenty three percent of infants who had received rhSOD3 treatment had respiratory illness severe enough to warrant treatment with asthma medications, compared to 36% of placebo controls (77). The effect was most significant in the most premature infants (less than 27 weeks gestational age) in whom 19% of infants that received rhSOD3 required asthma medications compared to 42% of controls. Additionally, there was a 44% decrease in the number of required hospitalizations and a 55% decrease in emergency room visits by the second year of life in the rhSOD3 group (77). This study also highlights the importance of extended follow-up in determining appropriate strategies for preventing BPD, as BPD has important long-term sequelae (74).

The same study cohort was reanalyzed for the effect of rhSOD3 treatment on ROP development. Overall, no significant differences in ROP development were noted in the rhSOD3 vs. the placebo group. However, when stratified according to level of prematurity, infants less than 26 weeks gestational age and less than 25 weeks gestational age had reduced ROP by 22 and 53%, respectively. Further, ROP severity greater than stage 2 developed in only 25% of the rhSOD3 treated group compared to 42% of controls (78). A protective effect of SOD3 in retinal vascularization is also suggested by studies using animal models of ROP. Hyperoxia significantly reduces retinal SOD3 levels in animal models of BPD and ROP. Transgenic mice that overexpressed SOD3 were exposed to 0.75 FiO_2_ for 5 days; these mice had significantly fewer extraretinal neovascular tufts than controls with normal SOD3 levels (79). Overall, these studies suggest a possible mechanistic link of oxygen-related injury in BPD and ROP that is related to reduced amounts of SOD3.

## Vitamin A

Vitamin A is important for proper lung and eye development, where it controls endothelial cell proliferation necessary for angiogenesis, among other functions (80, 81). Studies of vitamin A supplementation have been a major clinical focus in attempts to reduce BPD and ROP development and severity. Preterm infants are deficient in vitamin A because most of its placental transfer occurs during the third trimester of pregnancy (Figure 1). The low plasma vitamin A concentrations in these infants during the first weeks of life are associated with development of BPD (80, 82). The impact on vessel development in the lungs and eyes relates partly to the role of its active metabolite, retinoic acid, which inhibits VEGF and TGFβ expression while enhancing endothelial cell VEGFR2 signaling (80, 82, 83) connecting Vitamin A regulation and effects to the mechanisms discussed in relation to pathways shown in Figure 2 and discussed in section on TGFβ. Vitamin A also increases the number of mature alveoli (84). In a rat model of fetal lung hypoplasia, maternal vitamin A supplementation during pregnancy stimulates fetal sacculogenesis, and postnatal alveologenesis of the hypoplastic fetal/neonatal lungs (84). As lung angiogenesis and alveologenesis appear mechanistically intertwined, the potential impact of vitamin A on BPD development would appear to be significant. However, despite clear demonstration of its important functions in animal models, clinical trials of vitamin A deficiency in preterm infants during their first postnatal weeks using parenteral administration of 5,000 IU thrice weekly for the first 28 days of life showed disappointingly modest decreases in the incidence of BPD and death (84). Further, no such positive effect was seen in long-term follow-up (82).

Vitamin A supplementation of premature infants during the first weeks of life improved retinal function (defined as retinal rod sensitivity), likely through its essential role in regulating the formation of retinal photosensitive visual pigment and not from promoting the completion of retinal vasculogenesis (81, 85). In a rat model of ROP, treatment with retinoic acid significantly decreased VEGF levels compared to untreated ROP rats (83). This again shows a mechanistic link between Vitamin A and pathways depicted in Figure 2. A meta-analysis of three clinical studies found that vitamin A supplementation in preterm infants resulted in a trend toward a lessening of ROP, but this change was not significant (81).

Thus, like the clinical studies of BPD, currently the outcomes of studies of vitamin A supplementation to reduce ROP are also disappointing. How vitamin A treatment can better ameliorate the development of both BPD and ROP is not yet clear. Questions on dose timing, dose size, delivery method and optimal therapeutic formulation need to be further studied. In addition, complementing vitamin A with other therapies such as rhSOD3 or nitric oxide may enhance both short and long-term clinical outcomes. In support of this concept, a recent retrospective study suggested that vitamin A and nitric oxide synergistically improved pulmonary and neurodevelopmental outcomes for extremely preterm infants (birth weight <749 g) (86). Unfortunately, the effect on ROP was not reported.

## Thrombospondin-1

Thrombospondin-1 is part of a family of matricellular calcium binding extracellular glycoproteins that regulate cell-cell and cell-matrix interactions and is mainly localized to small and medium sized vascular structures (13, 87). The protein contains a procollagen-like domain and several type I repeats; these act on endothelial and vascular smooth muscle cells to produce its anti-angiogenic activity (87). Although little research has addressed effects of thrombospondin-1 in BPD and ROP, evidence suggests that it is a component in the pathogenesis of both diseases. Thrombospondin-1 was measured in autopsy samples of lung tissue from ventilated preterm infants born at 25–27 weeks gestational age. Thrombospondin-1 was upregulated 5.5-fold compared to lungs from non-ventilated infants of similar gestational age. The increased expression was localized to the lung interstitium with thrombospondin-1 positive platelet abundance in vessels of the ventilated lungs but rare in the unventilated lungs (13). Increased expression of thrombospondin-1 in the interstitium of immature lungs exposed to hyperoxic or inflammatory stimuli in addition to local release and activation of thrombospondin-1 from platelets traversing the pulmonary microvascular bed is a reasonable mechanism for how thrombospondin-1 may contribute to the reduced angiogenesis that occurs in BPD, possibly connecting to pathways shown in Figure 3 via inflammatory signals.

In studying the effects of thrombospondin-1 in ROP, newborn Sprague Dawley rats were exposed to a 24-h cycle of 50 and 10% oxygen for 2 weeks to reproduce the retinal hyperoxia and hypoxia stages of ROP. When this exposure was followed by injection with thrombospondin-1 immediately or 3 days after transfer to room air, there was a thrombospondin-1 dose-dependent decrease in retinal neovascularization in the rats who were injected immediately after transfer to room air compared to control rats (87). While very few human studies address thrombospondin-1 and its effect on ROP, based on these rat models of ROP discussed above, there is a potential role for this protein in angiogenic development and maturation warranting future focus for research as a potential mediator in BPD and ROP.

## Pigment epithelium derived factor

Pigment Epithelium-Derived Factor (PEDF) is a neuro-protective growth factor with significant anti-angiogenic properties; thus, its mechanism of action in BPD or ROP is of interest. PEDF is named for the retinal pigment epithelium cell layer where it was originally identified. Subsequent studies showed that PEDF is produced by epithelial cells in numerous organs and directly counteracts VEGF-induced neovascularization (88). A mouse model of BPD involving continuous exposure to hyperoxia during the alveolarization stage found reduced VEGF and markedly increased PEDF levels in the lung. Use of a transgenic PEDF knockout mouse showed protection against hyperoxia-induced changes of simplified alveolarization and microvascular remodeling in the lung, indicating that oxygen induction of PEDF is sufficient to produce the pulmonary structural characteristics of this BPD model (89).

PEDF levels were elevated during the avascular stage of developing ROP in the retinas of neonatal rat pups cycled daily between hyperoxia and hypoxia (90) (Figure 1). The ratio of PEDF protein to VEGF protein was significantly increased up to 14 post-natal days, reflecting the increased PEDF protein amounts present in the avascular phase of ROP. Thereafter, as the avascular phase progressed to the proliferative phase, PEDF levels decreased, presumably removing the inhibition of VEGF activity such that at 18 post-natal days neovascularization occurred in an uncontrolled fashion leading to the final ROP phenotype (91). Different rat strains exhibit different oxygen susceptibility for ROP development. While VEGF and VEGFR2 elevation occurred in all strains subjected to neonatal hyperoxia/hypoxia cycles, PEDF was elevated only in the oxygen-sensitive strains that developed ROP (92).

Hyperoxia induces the expression of the matrix metalloproteinases MMP2 and MMP9 in mouse lung and retinal cells (93–95); PEDF is a target of the proteinase activity of both MMP2 and MMP9 (95) connecting PEDF regulation with the pathways shown in Figure 2 and with the effects of O_2_ exposure. Mice with a genetic depletion of MMP2 expression have reduced retinal neovascularization in a hyperoxia model of ROP, consistent with continued activity of PEDF secondary to a loss of its breakdown (94). The same study found no effect of MMP9 genetic depletion on oxygen-induced retinopathy. PEDF promotes metabolism of oxygen radicals by stimulating glutamine synthetase and l-glutamate/l-glutamate transporter levels in neonatal mice with oxygen-induced retinopathy (96). Modifying PEDF levels through intravitreal drug injections has been investigated as a possible approach to prevent ROP. For example, retinal injections of endostatin or Bevacizumab into neonatal mouse eyes immediately following 5 days of hyperoxia restored PEDF/VEGF ratios by increasing PEDF; these retinas did not develop the neovascular changes of ROP (97, 98). Further research focusing on Thrombospondin 1, PEDF and other anti-angiogenic factors could prove useful for developing a more integrative understanding of BPD and ROP pathogenesis and contribute to uniform clinical approaches in order to more physiologically balance vessel development and vessel stabilization.

## Are BPD and ROP pathogenically related? conclusions and suggestions for future directions

Although this review presents substantial evidence supporting a hypothetical link between BPD and ROP pathogenesis, there are clearly other potential influencing factors beyond what we have discussed that may be of significance in one or both diseases. The state of current understanding for such factors, in addition to the ones presented in this review, reflects the increasing need for further research. This review focused on primary angiogenic and angiostatic growth factors. Other factors which influence angiogenesis such as fibroblast growth factor 1 (FGF-1) and platelet derived growth factor (PDGF) were not discussed because they likely have regulatory effects on several processes besides angiogenesis. Nevertheless, these also should be considered as potential candidates for mechanistic links between BPD and ROP (99–103). We have also not discussed specific inflammatory factors. While generally not primary regulators of angiogenesis, mediators of inflammation may indeed influence angiogenesis through regulation of the factors we discussed. Inflammatory regulators described in the pathogenesis of both BPD and ROP include monocyte chemotactic protein 1 (MCP-1/CCL2), macrophage inflammatory protein 1 alpha (MIP-1α/CCL3), interleukin 6 (IL-6) (5, 6, 104–108), and macrophage inhibitory factor (109–111). Finally, factors such as arginase-2, NADPH oxidase and erythropoietin are also implicated in both BPD and ROP (107, 112–118).

This review has explored the possibility of a mechanistic overlap in the development of BPD and ROP through the process of abnormal regulation of angiogenesis. No single angiogenic or angiostatic factor can fully account for the pathogenesis of either disease, yet many factors appear involved in some capacity. Further, current literature does not allow connecting the vascular factors discussed into one road map for the lung and eye alone or together for a map for normal or abnormal vasculogenesis in both organs. However, this review brings together information that may be useful for identifying potential candidates to consider for therapies to simultaneously decrease BPD and ROP development. It also emphasizes the need for further work to more fully elucidate the roles and timing of many of these factors in BPD and ROP. Finally, although not explicitly discussed in this review, the current studies of BPD and ROP in human and animal models are hampered by the lack of a clear consensus regarding the optimal animal model, including the specific approach to oxygen exposure, lack of a model of both diseases simultaneously, as well as the lack of agreement regarding the best clinical definitions of both diseases (119–121). Unfortunately, many study results are difficult to compare or use to extrapolate information that can be safely tested clinically after preterm birth (11). This review documents an abundant overlap of evidence that factors which dysregulate angiogenesis do so in both BPD and ROP. From this we propose that research approaches designed to simultaneously address potential common pathogenic mechanisms are likely to shed new light on both diseases and lead to a more holistic approach to preventing or treating these severe complications of prematurity.

## Author contributions

MV is Co-senior author with HN and MV contributed to the development of the topic for the review, aided, and advised AS in research and critical review of the literature, preparation, and organization of the manuscript. AS researched and provided the significant body of material reviewed and aided in the decision of data and information to be included in the manuscript as well as playing a significant role in preparing and revised the manuscript. CD aided in preparation, writing, and revision of the manuscript and contributed ideas on topics to be included in the review. HN is Co-senior author with MV and significantly contributed to the critical review of the literature to be included in the manuscript, as well as to guidance in preparation and organization of the manuscript.

### Conflict of interest statement

The authors declare that the research was conducted in the absence of any commercial or financial relationships that could be construed as a potential conflict of interest.

## Abbreviations

αSMA: Alpha smooth muscle actin
Ang1: Angiopoietin
Ang2: Angiopoietin 2
BAL: Bronchioalveolar lavage
BPD: Bronchopulmonary dysplasia
eNOS: Endothelial nitric oxide synthase
IFNγ: Interferon gamma
IL-6: Interleukin 6
IGF-1: Insulin like growth factor 1
IGFBP-2: Insulin like growth factor binding protein 2
IGFBP-3: Insulin like growth factor binding protein 3
IGFBP-4: Insulin like growth factor binding protein 4
IGFBP-5: Insulin like growth factor binding protein 5
IP-9and IP-10: Interferon γ-inducible 10-kDa proteins 9 and 10
MIP-1α/CCL3: Macrophage inflammatory protein 1 alpha
MIF: Macrophage Inhibitory Factor
MMP2: Matrix metalloproteinase 2
MMP9: Matrix metalloproteinase 9
MCP-1/CCL2: Monocyte chemotactic protein 1
NO: Nitric oxide
PMA: Post-menstrual age
PAPP-A: Pregnancy associated plasma protein A
ROP: Retinopathy of prematurity
sFlt-1: soluble fms-like tyrosine kinase-1
sTie2: Soluble Tie2
TGFβ: Transforming growth factor beta
VEGF: Vascular endothelial growth factor
VEGFR1: Vascular endothelial growth factor receptor 1
VEGFR2: Vascular endothelial growth factor receptor 2.
